# Valuation of the EQ-5D-Y-5L Using DCE Methods That Account for Nonlinear Time Preferences

**DOI:** 10.1177/0272989X251407950

**Published:** 2026-01-13

**Authors:** Alice Yu, Bram Roudijk, Peiwen Jiang, Richard Norman, Rosalie Viney, Deborah Street, Nancy Devlin, Mulhern Brendan

**Affiliations:** Centre for Health Economics Research and Evaluation (CHERE), University of Technology Sydney; EuroQol Research Foundation; Centre for Health Economics Research and Evaluation (CHERE), University of Technology Sydney; School of Population Health, Curtin University; Centre for Health Economics Research and Evaluation (CHERE), University of Technology Sydney; Centre for Health Economics Research and Evaluation (CHERE), University of Technology Sydney; Melbourne School of Population and Global Health, University of Melbourne; Centre for Health Economics Research and Evaluation (CHERE), University of Technology Sydney

**Keywords:** discrete choice experiment, EQ-5D-Y-5L, valuation study, nonlinear time preferences, pediatric HRQoL

## Abstract

**Objectives:**

Discrete choice experiment (DCE) methods that account for nonlinear time preferences have been tested in adult EQ-5D instruments but have yet to be tested for the valuation of EQ-5D-Y instruments. The aims of this study were to test the feasibility of using DCE methods that model nonlinear time preferences for the valuation of the EQ-5D-Y-5L as well as to explore the impact of the perspective adult respondents are asked to take.

**Methods:**

A representative Australian adult general population sample completed an online survey that included 15 DCE split triplet tasks. Depending on arm assignment, respondents were asked to imagine themselves or a 10-y-old when choosing between health states. A Bayesian efficient design was used to construct DCE tasks; the design was updated 3 times. Data were analyzed using correlated mixed logit models with exponential discounting.

**Results:**

There were 955 and 947 respondents in the “self” and “10-y-old” arms, respectively. When nonlinear modeling is used, there is evidence of discounting in the “self” (17%) and “10-y-old” (15%) perspective. Avoiding the experience of pain and discomfort were most important in both arms. When imagining a 10-y-old, rather than “self,” respondents considered being worried, sad, or unhappy to be more important. Sensitivity analysis revealed that nonparents considered a higher number of health states to be worse than dead when imagining themselves.

**Conclusions:**

This is the first study to use a nonlinear DCE approach in the valuation of the EQ-5D-Y-5L and in pediatric health-related quality of life more generally. Nonlinear modeling methods were found to be suitable for the valuation of the EQ-5D-Y-5L.

**Highlights:**

- There is evidence that modeling for nonlinear time preferences is suitable for the valuation of adult health-related quality of life (HRQoL). It is unknown how time preferences affect the valuation of pediatric instruments, such as the EQ-5D-Y-5L, and whether this differs when adults are asked to imagine “self” versus a “10-y-old.”
- There was evidence of nonlinear time preferences when adult respondents value health states for a 10-y-old using a discrete choice experiment (DCE) that included a duration attribute. Perspective was a strong driver of estimating states worse than dead: 42% of health states were considered worse than dead for a 10-y-old as opposed to 26% when respondents imagined themselves.
- Nonlinear DCE methods are feasible for the valuation of the EQ-5D-Y-5L and have advantages compared with the use of time tradeoff in valuing child HRQoL. Future studies can test whether nonlinear modeling methods are suitable for other pediatric HRQoL instruments.

## Introduction

The EQ-5D is the most common health-related quality of life (HRQoL) instrument cited in national health technology assessment (HTA) guidelines.^1^ The EQ-5D-Y-3L was developed for use in pediatric populations in 2010.^2^ Expanding on the EQ-5D-Y-3L, the 5-level version, the EQ-5D-Y-5L, has recently been approved by the EuroQol Group.^3,4^ Evidence suggests that the EQ-5D-Y-5L has improved construct validity and responsiveness^5,6^ compared with the EQ-5D-Y-3L. Reflecting the growth in use of the adult EQ-5D-5L, the EQ-5D-Y-5L is likely to become more widely used, and there is work underway to inform a valuation protocol. This has been underpinned by debate about the perspective and type of valuation methods to use in the valuation of pediatric HRQoL instruments.^7^

For adult populations, detailed guidelines concerning HRQoL values for use in economic evaluation are available from major HTA agencies.^8–11^ However, many emerging health technologies target children and adolescents.^12^ Major HTA agencies have generally used evidence based on valuations of adult HRQoL in pediatric-specific technology assessments.^7,13^ This is problematic, as adults have been found to value HRQoL differently for themselves and pediatric populations.^14,15^ There is also evidence to suggest that there are fundamental differences in value sets for the EQ-5D-Y-3L compared with the adult EQ-5D instruments.^16^ Hence, it is questionable whether it is appropriate for HTA agencies to use adult values for HRQoL to reflect pediatric HRQoL.

In terms of valuation method, there is emerging evidence that a discrete choice experiment (DCE) with duration approaches, in which respondents consider health profiles including both a health state and an accompanying life expectancy, have been found to be feasible in both adults and adolescents.^17–22^ Separately, there is evidence that constant proportionality does not hold and that models that allow for time discounting or nonlinear time preferences (i.e., events in the future to be valued less than events closer to the present) provide a better reflection of respondent health preferences.^23^ In such models, it assumed that future life-years may be more or less important than the present. In practice, a model for the DCE data is estimated that allows such preferences to be captured, if they exist, and collapse to linear preferences otherwise.^24^ There is evidence of nonlinear time preferences in adult instruments.^25,26^ Jonker et al.^24^ estimated an exponential discount rate of 5.7% and a hyperbolic discount rate of 16.5% in the valuation of the SF-6D.

DCE studies that account for nonlinear time preferences often use a DCE with duration approach, in which the third health state is always full health for a shorter duration.^27–29^ When valuing EQ-5D-Y-5L using DCE with duration approaches, each choice option in the DCE task consists of a health state described using the 5 dimensions of the EQ-5D and an additional attribute for duration, giving 6 attributes in total. Following the model in Jonker et al.,^24^ latent scale disutilities are estimated for each of the 5 EQ-5D-Y-5L dimensions. Furthermore, a scaling parameter is estimated that reflects tradeoffs between the EQ-5D-Y-5L and the duration of life, to anchor the disutility weights on the quality-adjusted life-year (QALY) scale. This means that the zero point of the QALY scale is interpolated. In the QALY model, we assume that full health has a value of 1 and a death a value of 0. A disutility of 1 therefore reflects the distance between full health and death. Those health states that receive a disutility larger than 1 therefore have negative utility values, reflecting that (most) respondents would rather live shorter durations in such states than live longer but in those health states. In each choice task, respondents choose between 2 health states at a time. Each health state is assumed to start at the same time, and after the described duration, the individual dies. The net present value for each of the lives is calculated, and a discount rate that best describes the choice behavior of respondents is estimated. Since disutilities are modeled, there is no negative, only disutilities that are not classified into better or worse than dead. Instead, the zero point on the scale is interpolated using a logit model. The discounting plays a role in interpolating the zero point, as it affects the scale of the model identified.

Using a single-valuation approach may have advantages over more complex protocols that combine data from different methods (e.g., time tradeoff [TTO] tasks and DCE choice tasks without duration,^28^ for the EQ-5D-Y-3L). For instance, it may mean respondents are required to complete fewer tasks. The TTO valuation of pediatric HRQoL has been problematic, with adult respondents unwilling to trade children’s life-years, leading to relatively high values for pediatric HRQoL.^12,16^ Recent evidence also supports the notion that in terms of scale and importance of dimensions, valuation using TTO and DCE choice tasks with duration produce values with similar values as DCE choice tasks, at least for the adult EQ-5D-5L.^26^ This suggests that different methods of valuation (and anchoring) have the potential to capture similar concepts and lead to consistent results (in the absence of a gold standard measure of preferences).

It is unknown how time preferences might affect adult valuation of pediatric health states. So far, valuation studies of nonlinear time preferences have been undertaken for only adult instruments in which adults value health states from a “self” perspective. These responses may be influenced by personal time preferences (as well as preferences for the HRQoL dimensions). In the case of adults valuing child HRQoL, it is unknown whether preferences for dimensions and duration are the same.

We report a study valuing the EQ-5D-Y-5L using a DCE modeling approach that models nonlinear time preferences among the Australian adult population.

The aims of this study are as follows:

1. to explore whether and how adult respondents discount time when valuing EQ-5D-Y-5L health states,
2. to understand the impact of asking adult respondents to imagine themselves versus a 10-y-old on the values for EQ-5D-Y-5L obtained using nonlinear DCE with duration, and
3. to explore whether adult respondents value EQ-5D-Y-5L health states differently if they are a parent/caregiver or someone without children (nonparent).

## Methods

### Recruitment and Study Arms

This study recruited a representative sample of the adult Australian population in terms of age, gender, and state. There were 2 arms in this study, with arm 1 collected prior to arm 2. This was to allow for the design update process required for each arm (see the “DCE Choice Tasks Update Process” subsection below). In arm 1, respondents were asked to imagine themselves as they completed the choice tasks. In arm 2, respondents were asked to imagine a 10-y-old child. Respondents were recruited from a major panel provider in Australia, PureProfile (https://www.pureprofile.com/).

### Survey Overview

In the survey, information related to demographics, health questions, and follow-up questions about the DCE choice tasks were collected. A detailed survey overview can be found in Appendix A, and a copy of the survey is available on request. Maths in Health (https://www.mathsinhealth.com/) programmed and hosted the survey.

### DCE Choice Tasks

Prior to completing the DCE choice tasks, respondents were shown a short text and picture tutorial over 4 pages, available in Appendix B. Each respondent was shown 15 DCE choice tasks (with 2 choices per task), in which each DCE choice task entailed considering 3 health states. Each health state was described by a combination of EQ-5D-Y-5L dimension levels and a duration attribute that described the number of years the health state would be experienced before dying. Duration levels used were 0.25 (3 mo), 0.5 (6 mo), and all integers from 1 to 15 y. The overlap of 2 dimensions was also allowed in DCE choice tasks, to increase the ease of decision making for respondents.

Each choice task is divided into 2 parts. In part 1, respondents were asked to choose between health states A and B, where the same duration is used for each state (so the tradeoff focuses on HRQoL). In part 2, respondents were asked to choose between health states B and C (irrespective of whether A or B was chosen for part 1), in which health state C is always full health (i.e., “no problems” on each dimension) for a shorter duration than that of health states A and B. This is the split triplet choice format that has been used in previous studies.^25,26,29^

**Figure 1:**
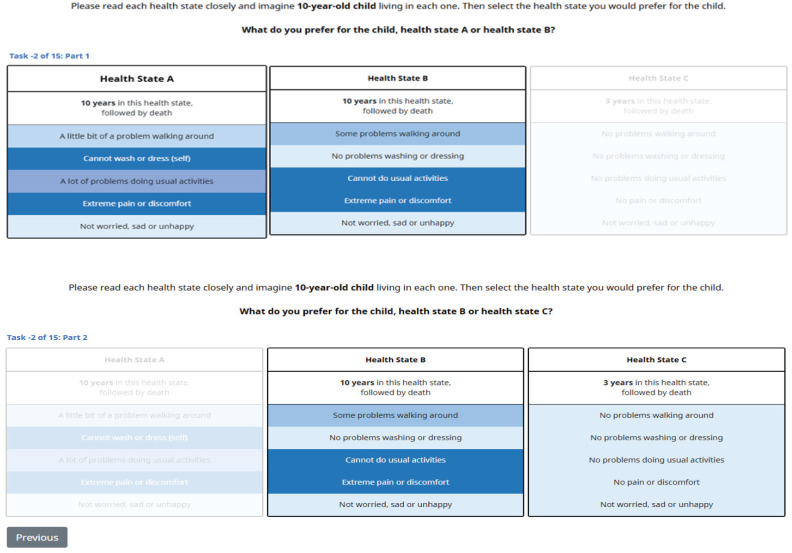
Example choice task with 10-y-old perspective: part 1 and 2.

Figure 1 provides an example choice task. To help respondents differentiate between dimension levels, a blue gradient has also been used to indicate the severity of the level, with a darker shade of blue indicating a more severe level. The use of color coding has been used in previous studies with similar choice tasks.^26,28,29^

### DCE Choice Tasks Update Process

A Bayesian efficient design was used to construct the DCE choice tasks. These were constructed in the time preferences corrected QALY design (TPC-QD) software with customized code that has been used in previous studies.^24,30^ Details of the design optimization process can be found in Appendix C.

The Bayesian efficient design requires priors for the model parameters. Initial priors for the 20 interaction terms between EQ-5D-Y-5L dimension levels and duration were based on the anchored coefficients from the Australian population EQ-5D-5L value set^31^ to create the first design of 150 DCE choice tasks. Upon collecting this sample of approximately 200 respondents, the data were analyzed and a new design generated using the estimates as priors. Subsequently, a new sample of approximately 200 respondents was collected, and the combined data were analyzed, with the results serving as priors for the updated design. This was repeated until the results of the analysis roughly matched the previously used priors for the last design update or until the data collection was completed. Overall, 1,000 respondents were planned to be collected per arm.

### Data Analysis

#### Quality checks

The final sample of respondents was determined based on a set of data quality criteria. Respondents were included only if they consented to participate and completed the whole survey without quality issues being flagged. Respondents were excluded if there was a likelihood that the response was from a bot. This was done by capturing the reCAPTCHA v3^i^ Google scores 4 times. Google reCAPTCHA v3 works as follows. First there is a piece of code that captures the mouse activity on the screen. Based on that information, there is a model that estimates the probability of that activity being from a human or from a bot. The model output is a score ranging between 0 and 1, where 0 would mean that the screen activity was from a bot and 1 would mean that the screen activity was from a human. It should be noted that the model output is never exactly 0 or 1, so the prediction is never with 100% certainty. In this study, mouse screen activity was captured 4 times, and if in any of those 4 instances the score was lower than 50%, then it was assumed the respondent was a bot and was excluded.

Respondents were also excluded if they had a median response time of less than 12.5 s per DCE choice task (i.e., choice between health state A versus B and B versus C). This was guided by what has been used in previous studies.^26^ Largely motivated by data quality issues experienced during the initial stages of recruitment (see Appendix D), respondents also had to pass an inverse visual analog scale (VAS) traffic light task, in which they are asked to order red, orange, and green traffic lights along a VAS scale in reverse order (i.e., where 100 means “cars must stop” and 0 means “cars can go”).

As a further consistency check, respondents who said, at the beginning of the survey, that they had children were also asked that question after the completion of the DCE choice tasks. They were also asked to restate how many children they had and the age of their child or oldest child at their last birthday. For respondents who indicated they had no children at the beginning of the survey, they were instead asked about their age on their last birthday again, after the completion of the DCE choice tasks.

#### Modeling nonlinear time preferences

The initial prior for parameter “perfect health” was set to have a mean of 1 and a standard deviation of 0.2. For the discount rate parameter, an initial prior value of 7.5% (±2%) was selected. The initial prior for the discount rate parameter was motivated by a need to provide broad enough coverage for a first estimate. For each design update, priors for the parameters perfect health and the discount rate were based on model parameter estimates. For the first update, a conditional logit model with a discount rate was used. Subsequent data collections were sufficiently powered to estimate mixed logit models (MXL), so for subsequent updates, MXL estimates were used as point priors.

The final models were based on data from all respondents in each arm who passed the data quality checks. Correlated MXL models were estimated on the final data. The correlated MXL model collapses to an uncorrelated MXL if no statistically significant correlation is present, which in turn collapses to a conditional logit model if there is no statistically significant heterogeneity between respondents. Standard MXL models were also estimated, assuming linear time preferences, using the garbage_mxl command in STATA^32^; this is available in Appendix E.

Analyses were conducted in OpenBUGS.^33^ The model specification is based on Jonker et al.,^24^ which allows for linear time preference as a special case. For the MXL model, utility *U* for individual i for alternative n in choice set j is specified as

(1) $$U_{\mathrm{inj}}=\left(\beta_{i}X_{\mathrm{inj}}\right)\mathrm{NP}V_{\mathrm{inj}}+\varepsilon_{\mathrm{inj},}\beta_{i}\sim \mathrm{MVN}(\beta ,\Sigma )$$

where $\beta_{i}$ are the preference parameters associated with individual i that are assumed to be multivariate normal distributed with population mean β and covariance matrix Σ is the attribute levels shown to individual i in alternative n of choice set j . The net present value $\mathrm{NP}V_{\mathrm{inj}}$ is the sum of the present value of future life-years ( $\mathrm{TIM}E_{\mathrm{inj}}$ ). In this case, the net present value, $\mathrm{NP}V_{\mathrm{inj}}$ , is discounted using the standard exponential function. The standard exponential function allows for linear time preference as a special case when the discount rate (*r*) is equal to zero. This can be expressed as

(2) $$\mathrm{NP}V_{\mathrm{inj}}=\mathrm{TIM}E_{\mathrm{inj}},\mathrm{if}\;r=0.$$

The more general case in which the discount rate is not zero can be expressed as

(3) $$\mathrm{NP}V_{\mathrm{inj}}=((1-e^{\left(-r\right)\mathrm{TIM}E_{\mathrm{inj}}}))/(e^{r}-1)),\mathrm{if}\;r\neq 0.$$

#### Reporting of results

Results are reported on the QALY scale (i.e., a scale anchored at 1 for full health and 0 for dead) with the associated 95% confidence intervals. The QALY scale values were obtained by dividing the estimate of the mean $\hat{\beta }$ by the first element of $\hat{\beta }$ , that is, the perfect health intercept, ${\hat{\beta }}_{1}$ . This can be expressed as

(4) $$\mathrm{QAL}Y_{\mathrm{decrement}}=\hat{\beta }/{\hat{\beta }}_{1}.$$

The QALY scale values were estimated directly in OpenBugs^33^ along with the raw parameter estimates. Raw parameter estimates are available in Appendix G.

Sensitivity analysis was undertaken to determine how results differ if arms are separated into respondents who identified as parents/caregivers versus nonparents. Correlated MXL models were estimated separately for parents and nonparents in each arm. Selected health state values were then calculated for comparison purposes.

## Results

### Final Sample for Analysis

A summary of respondent numbers by round of data collection can be found in Appendix F. The decision process for the final sample is summarized in Table 1. In total, 955 respondents in the “self” arm and 947 respondents in the “10-y-old” arm were included for analysis. Respondents who provided inconsistent responses to the repeated question about their age or their parental status were excluded from the final analysis.

**Table 1:** Decision Process to Final Sample

Decision Process to Final Sample	“Self” Arm		“10-y-Old” Arm	
	No.	% Passed^a^	No.	% Passed
1. Completed whole survey	3,274		3,824	
2. Passed Google reCAPTCHA v3 test^b^	1,268	39%	1,782	47%
3. Passed speed test (>12.5 s/choice task)	1,004	31%	1,351	35%
4. Passed inverse VAS traffic light test (traffic light test)	Error^c^		1,011	26%
5a. Passed age consistency check (nonparents)	994	30%	1,005	26%
5b. Passed parental status/children consistency check (parents)	955	29%	947	25%

VAS, visual analog scale.

a Percentage who passed each quality check relative to those who completed the whole survey.

b The reCAPTCHA v3 test is repeated 4 times; the respondent is excluded if, in any of the 4 instances, the score was lower than 50%, where 0 means 100% bot and 1 means 100% human.

c See Appendix F.

In total, choice information was from the initial DCE choice task design plus 3 updates of the design information, resulting in 600 choice tasks (4 × 150 choice sets) per arm. The aim was for approximately 200 respondents per round of data collection. Due to a technical error during data collection, 243 of 1,004 respondents who did not pass the inverse VAS traffic light test (traffic light test) were mistakenly recorded as having passed. This was discovered only after data collection had ended; thus, these respondents were included as part of the update of the DCE choice tasks.

### Respondent Characteristics

A table summarizing the sample demographics can be found in Appendix H. Respondents were generally representative of the Australian population in terms of gender and state/territory. There was slight underrepresentation of respondents between the ages of 18 and 24 y, largely due to difficulties recruiting men in this age group. Most respondents (85% in both arms) spoke English as their main language at home. Sixty-three percent and 64% of respondents in the “self” and the “10-y-old” arm, respectively, were parents/caregivers; this is comparable to the number of families with children in Australia at 59.6%.^34^ The proportion of respondents with a bachelor’s degree or higher was 47% in “self” arm and 44% in “10-y-old” arm compared with 26% within the Australian population.^35^ About half of respondents lived in a household that earned AUD 1,500 or more per week, compared with the Australian median household income of AUD 1,770 per week.^36^ Most respondents described their own health as good or better than good: 74% in the “self” arm and 77% in the “10-y-old” arm. Most respondents would also describe themselves as usually happy or very happy: 67% in the “self” arm and 69% in the “10-y-old” arm.

### DCE Choice Task Difficulty

A summary of how respondents rated DCE choice tasks can be found in Appendix I. Chi-square tests were performed to test for differences by arm. It was found that respondents in the “10-y-old” perspective arm were significantly more likely to find DCE choice tasks challenging than those completing the tasks from their own perspective. They were more likely to find it difficult to tell the difference (*P* < 0.01) and choose (*P* < 0.01) between health states and generally found the choice tasks more difficult (*P* < 0.01).

### Model Results

A summary of QALY scale value estimates can be found in Table 2. The estimated discount rates are substantially larger than 0%, indicating that the assumption of nonlinear time preferences does hold. In both arms, all QALY scale values were significant at the 95% confidence level with no disordering of decrements in QALY scale values. This indicates that respondents had ordered preferences and would significantly prefer the baseline (i.e., level 1), compared with the more severe levels of 2 to 5 in each dimension.

**Table 2:** Mixed Logit Model Parameter Estimates: Decrements in QALY Scale Values

QALY Scale Value	“Self” Arm (*n* = 955)				“10-y-Old” Arm (*n* = 947)			
	Mean	SD	L95%CI	U95%CI	Mean	SD	L95%CI	U95%CI
MO2	−0.06	0.01	−0.07	−0.04	−0.06	0.01	−0.08	−0.05
MO3	−0.09	0.01	−0.1	−0.07	−0.09	0.01	−0.11	−0.07
MO4	−0.22	0.01	−0.24	−0.19	−0.23	0.01	−0.25	−0.2
MO5	−0.43	0.02	−0.47	−0.39	−0.35	0.02	−0.39	−0.32
SC2	−0.05	0.01	−0.06	−0.04	−0.02	0.01	−0.04	−0.01
SC3	−0.07	0.01	−0.08	−0.05	−0.06	0.01	−0.08	−0.04
SC4	−0.2	0.01	−0.22	−0.18	−0.15	0.01	−0.17	−0.13
SC5	−0.41	0.02	−0.44	−0.37	−0.27	0.02	−0.3	−0.24
UA2	−0.03	0.01	−0.04	−0.01	−0.03	0.01	−0.05	−0.02
UA3	−0.06	0.01	−0.07	−0.04	−0.07	0.01	−0.09	−0.05
UA4	−0.16	0.01	−0.19	−0.14	−0.19	0.01	−0.21	−0.17
UA5	−0.3	0.01	−0.33	−0.27	−0.31	0.02	−0.35	−0.28
PD2	−0.05	0.01	−0.07	−0.04	−0.09	0.01	−0.11	−0.07
PD3	−0.09	0.01	−0.11	−0.08	−0.14	0.01	−0.16	−0.12
PD4	−0.29	0.01	−0.32	−0.26	−0.44	0.02	−0.49	−0.4
PD5	−0.58	0.03	−0.63	−0.53	−0.89	0.04	−0.98	−0.81
WSU2	−0.06	0.01	−0.08	−0.05	−0.11	0.01	−0.13	−0.09
WSU3	−0.17	0.01	−0.19	−0.15	−0.26	0.02	−0.3	−0.24
WSU4	−0.24	0.01	−0.27	−0.21	−0.39	0.02	−0.43	−0.35
WSU5	−0.39	0.02	−0.43	−0.35	−0.6	0.03	−0.66	−0.54
Discount rate	0.17	0.01	0.15	0.18	0.15	0.01	0.13	0.16
Log likelihood	−9,982	109.3	−10,200	−9,768	−9,813	119.4	−10,040	−9,576

CI, confidence interval; MO, mobility (walking around); PD, pain and discomfort; QALY, quality-adjusted life-year; SC, self-care (washing or dressing); SD, standard deviation; WSU, worried, sad, or unhappy; UA, usual activities.

**Figure 2:**
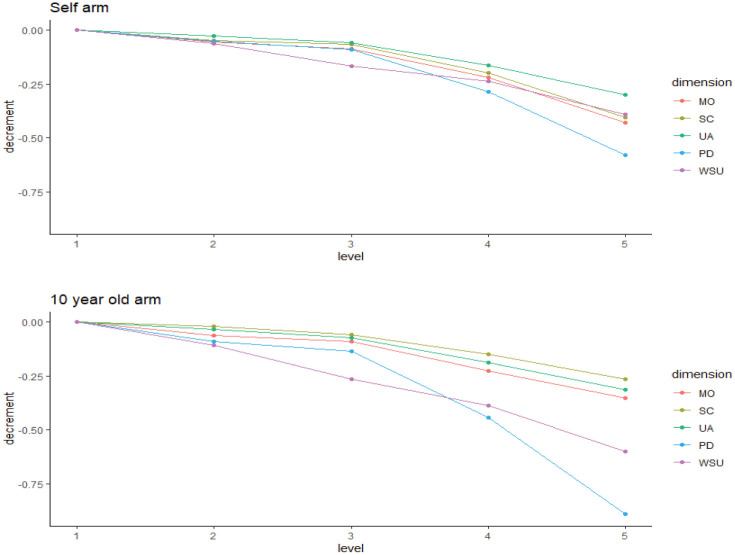
Plot of quality-adjusted life-year scale value decrements by EQ-5D-Y-5L dimension in the “self” and “10-y-old” perspective arms.

Figure 2 provides a visual representation of QALY scale value decrements. The dimension PD (i.e., pain and discomfort) was most important to respondents in both arms. However, PD was associated with higher decrements in QALY scale values when imagining a 10-y-old. After PD, feelings of worried, sad, or unhappy were also relatively more important to avoid for respondents when imagining a 10-y-old, whereas when imagining self, mobility was relatively more important to avoid.

It was noted that for the “self” arm, a change from level 2 to 3 (i.e., “a little bit” to “quite worried, sad, or unhappy”) had a higher value decrement than changes from levels 2 to 3 on other dimensions. Respondents who were asked to imagine a 10-y-old generally had higher value decrements when moving from level 3 to 5 (quite/really/extremely worried, sad, or unhappy) compared with those who were asked to imagine themselves. Feelings of being worried, sad, or unhappy were seen as more serious for a 10-y-old when compared against the same feelings for respondents themselves.

Table 3 provides summary statistics of the calculated health state values from the QALY scale value decrements.

**Table 3:** Summary of EQ-5D-Y-5L Health State Values Produced by Adults’ Responses to Nonlinear Discrete Choice Experiment with Duration Tasks, by Perspective Adopted (“Self” v. “10-y-Old Child”)

State	“Self” Arm	“10-y-Old” Arm
11111	1	1
11211	0.97	0.97
12111	0.95	0.98
22222	0.75	0.68
33333	0.53	0.38
44444	−0.11	−0.39
55555	−1.1	−1.42
% States with values <0	26%	42%

The value of the worst state (i.e., 55555), was lower for the 10-y-old child arm at −1.42 as opposed to −1.10 for the “self” arm. It was also of note that more health states were estimated as “worse than dead” (i.e., had values < 0) when respondents were asked to imagine a 10-y-old (42%) compared with when they were asked to imagine themselves (26%).

### Sensitivity Analyses

#### “Self” arm: Removing respondents who failed the VAS traffic light test

As mentioned earlier, during recruitment, 243 of the 955 respondents in the “self” arm were mistakenly included as part of the DCE choice task updates, and results reported include these respondents as well. A correlated MXL model was estimated excluding these 243 respondents (raw parameter estimates are available in Appendix J). Results were consistent with those in which all 955 respondents are used, suggesting the inclusion of those who failed the traffic light test did not have a major effect on results.

#### Parents versus nonparent valuation of health states

There was an overrepresentation of parents/caregivers (>60%) in both arms. Key health state utilities, percentage of health states with values <0, and discount rate for each group are summarized in Table 4.

**Table 4:** “Self” Arm: EQ-5D-Y-5L Health State Values of Parents versus Nonparents

Health State Value	“Self” Arm		“10-y-Old” Arm	
	Parents (*n*= 611)	Nonparents (*n* = 344)	Parents (*n* = 592)	Nonparents (*n* = 354)
11111	1	1	1	1
11211	0.97	1.00	0.97	0.98
22222	0.77	0.75	0.68	0.73
33333	0.59	0.46	0.39	0.42
44444	0.00	−0.25	−0.37	−0.35
55555	−0.86	−1.48	−1.33	−1.48
% <0	17%	39%	39%	41%
Discount rate	24%	16%	20%	17%

Results were relatively similar for parents versus nonparents in the “10-y-old” arm. The greatest contrast is seen in results for the “self” arm. The length of the utility scale was much shorter for parents compared with nonparents. This is also reflected in nonparents considering a higher number of health states to be worse than dead compared with parents in the “self” arm. Discount rates were significant at the 5% level for all 4 groups, indicating the presence of discounting regardless of perspective when valuing the EQ-5D-Y-5L.

#### Comparison of results with EQ-5D-Y-3L values

Results were also compared against the published Australian EQ-5D-Y-3L estimates.^37^ This was done by extracting 243 of the 3,125 health states that have the same labels as those from the EQ-5D-Y-3L. The EQ-5D-Y-3L uses 3 levels: no, some, and a lot/very, which corresponds to levels at 1 (no), 3 (some), and 4 (a lot of) in the EQ-5D-Y-5L. Scatterplots were then generated to compare against the “self” and “10-y-old” perspective, available in Appendix K. There appears to be good agreement in terms of distance between the EQ-5D-Y-3L value set and the EQ-5D-Y-5L values reported here using the “10-y-old,” but not in terms of scale. The opposite was true for the “self” perspective, with better agreement in terms of scale but more variation in terms of distance.

## Discussion

To our knowledge, this is the first study to apply nonlinear DCE methods to the valuation of pediatric HRQoL. When discount rates were directly estimated, the assumption of nonlinear time preferences held in both arms. Experience of pain and discomfort and feelings of being worried, sad, or unhappy were associated with higher QALY decrements for a 10-y-old, suggesting adults are more willing to accept shorter durations of life to avoid such health problems. However, other considerations could also affect these choices, such as the impact on the family of a child or that time in full health is less important for children.

Most notably, adult respondents were much more sensitive to a 10-y-old experiencing severe health problems, with many more health states considered worse than dead, compared with respondents who valued those same health states from their own perspective. This discrepancy could be partially attributed to the framing of the question as imagining a 10-y-old child (i.e., someone else) as opposed to asking respondents to imagine themselves as a 10-y-old child. Powell et al.^14^ in their study on valuing child and adolescent health, reported that participants were most comfortable responding for themselves and that most reported that their answers would be different if they were thinking about someone else. This contrasts with what has been found in TTO valuation of child health states,^12,16^ in which a reluctance to sacrifice a child’s life-years seems to dominate valuation of poor HRQoL. This is not seen when nonlinear DCE methods are used. Sensitivity analysis also suggests that being a parent may not be a relevant factor in valuing a child’s health but may be relevant when valuing your own health.

Results reported here contrast with characteristics of value sets for the EQ-5D-Y-3L, produced using the EQ-VT protocol.^38,39^ EQ-5D-Y-3L values, produced using a combination of latent scale DCE and TTO, have produced values for the worst health state much closer to 0, and the length of the value scale is therefore shorter.^40–42^ These differences could be due to a number of factors. The way duration is used to anchor value sets is different. The EQ-5D-Y-3L protocol uses composite TTO (cTTO) to anchor value sets, and nonlinear time preferences are not considered in the modeling. In cTTO, respondents are asked directly whether the experience of a state would be better or worse than being dead. This may keep the values of health states higher than when this question is not asked. Jakubczyk et al.^43^ noted that comparisons to immediate death reduces the number of health states considered worse than death, while comparisons in terms of prolonging life in a given health state increases it. This is supported by exploratory work in the nonlinear modeling context,^27,44^ in which anchoring to full health produces more health states considered worse than dead as compared with anchoring to immediate death. The type of DCE task shown to respondents also differs. The EQ-5D-Y-3L protocol requires respondents to complete DCE choice tasks without duration, whereas this study uses the format of DCE choice tasks with full health.

There is a difference in findings between the current study, valuing the EQ-5D-Y-5L and the characteristics of EQ-5D-Y-3L value sets^12^ including the recent EQ-5D-Y-3L value set for Australia,^37^ produced using an international protocol based on cTTO and latent scale DCE.^38^ This is in contrast to recent findings regarding adult HRQoL in which consistent results were found between the cTTO and nonlinear DCE methods used in the valuation of the EQ-5D-5L.^26^ The difference between cTTO (used to value EQ-5D-Y-3L) and nonlinear DCE values for child health states may arise because of adult respondents’ greater reluctance to trade off life-years in TTO tasks involving child health states.^16^ Further research is needed to investigate the reason for such differences for adult versus pediatric instruments. Future studies could also use nonlinear modeling methods in the valuation of the EQ-5D-Y-3L and compare against the current findings, to further explore the difference in values considered worse than death.

The differences in results between EQ-5D-Y-3L value sets and the current study also represent a challenge for users and decision makers who choose which value sets should be used in policy settings, and sensitivity analysis across value sets may be required in the evaluation of new technologies or in comparisons of population health. It is also important for HTA agencies to provide clearer guidance on the perspective to use in pediatric HRQoL instruments to encourage their use in economic evaluations of pediatric health technologies.

There are some limitations to note for this study. In the “self” arm, about 24% of respondents over the 4 rounds of data collection were accidentally included even though they did not pass one of the quality checks. Although results were very similar with and without the inclusion of these respondents, it must be acknowledged these respondents are still “embedded” in a way, as their responses were included in the DCE choice task update process. The VAS traffic light task may not be intuitive to all respondents and hence may be considered a very conservative (strict) quality control task. It was included in this study to address quality issues with data during the initial recruitment and data collection. In future valuation studies for pediatric HRQoL instruments, this quality control task may not be necessary or may be modified or replaced by other measures to ensure respondents are truly engaged.

Data quality in online samples is a concern. To address this in the study, the Google reCAPTCHA v3 test was repeated 4 times to detect and exclude potential bots. However, there is the possibility that this test may not have captured all bots. Our exclusion process included several additional quality checks, on top of the Google reCAPTCHA v3 test, which resulted in a high level of exclusions demonstrating the close consideration of data quality and provides confidence of the inclusion of only high-quality respondents in this study. One of the additional quality checks was using a repeat question of age for respondents who indicated they were not parents and a repeat question about their children if respondents indicated they were parents. The use of a different quality criteria question for parents versus nonparents is a potential limitation to this study.

It is also important to be cautious in interpreting the estimated discount rates. As this is the first study to use nonlinear modeling methods in the valuation of a pediatric HRQoL instrument, it is difficult to tell how valid the estimates were. More valuation studies using nonlinear modeling for pediatric instruments are needed for comparison. When compared against studies valuing adult HRQoL instruments, the current discount rates are not unusual. Jonker et al.^24^ reported 5.7% exponential and 16.5% hyperbolic discount rates in the valuation of the SF-6D. More recently, Pullenayegum et al.^44^ reported a discount rate of 23% in the valuation of EQ-5D-5L. Yu et al.^27^ illustrated how discount rates can be influenced by a range of factors including sample size, data quality, choice of anchor used in DCE with duration tasks, as well as the choice of DCE experimental design of choice tasks, finding discount rates ranging from 1% to 117%. The discount rates elicited from such tasks tend to be considerably higher than the discount rates typically applied in HTA. The implications of this apparent discord between individual time preference and the social time preference applied in HTA is unclear.

There is also some evidence to suggest that the discount rate captures not only time preferences but also some forms of heteroskedasticity, as the error term is assumed to be similar in size between choice tasks with different durations, which may not be realistic. In addition, the time preferences exhibited may be influenced by the nature of the task (choosing durations of survival) and by the range of durations presented.

This study provides evidence that nonlinear methods are feasible to use in the valuation of EQ-5D-Y-5L. Future studies can test whether nonlinear modeling methods are suitable for other pediatric HRQoL instruments.

## Supplemental Material

sj-docx-1-mdm-10.1177_0272989X251407950 – Supplemental material for Valuation of the EQ-5D-Y-5L Using DCE Methods That Account for Nonlinear Time Preferences Supplemental material, sj-docx-1-mdm-10.1177_0272989X251407950 for Valuation of the EQ-5D-Y-5L Using DCE Methods That Account for Nonlinear Time Preferences by Alice Yu, Bram Roudijk, Peiwen Jiang, Richard Norman, Rosalie Viney, Deborah Street, Nancy Devlin and Mulhern Brendan in Medical Decision Making

sj-docx-2-mdm-10.1177_0272989X251407950 – Supplemental material for Valuation of the EQ-5D-Y-5L Using DCE Methods That Account for Nonlinear Time Preferences Supplemental material, sj-docx-2-mdm-10.1177_0272989X251407950 for Valuation of the EQ-5D-Y-5L Using DCE Methods That Account for Nonlinear Time Preferences by Alice Yu, Bram Roudijk, Peiwen Jiang, Richard Norman, Rosalie Viney, Deborah Street, Nancy Devlin and Mulhern Brendan in Medical Decision Making

sj-docx-3-mdm-10.1177_0272989X251407950 – Supplemental material for Valuation of the EQ-5D-Y-5L Using DCE Methods That Account for Nonlinear Time Preferences Supplemental material, sj-docx-3-mdm-10.1177_0272989X251407950 for Valuation of the EQ-5D-Y-5L Using DCE Methods That Account for Nonlinear Time Preferences by Alice Yu, Bram Roudijk, Peiwen Jiang, Richard Norman, Rosalie Viney, Deborah Street, Nancy Devlin and Mulhern Brendan in Medical Decision Making

sj-docx-4-mdm-10.1177_0272989X251407950 – Supplemental material for Valuation of the EQ-5D-Y-5L Using DCE Methods That Account for Nonlinear Time Preferences Supplemental material, sj-docx-4-mdm-10.1177_0272989X251407950 for Valuation of the EQ-5D-Y-5L Using DCE Methods That Account for Nonlinear Time Preferences by Alice Yu, Bram Roudijk, Peiwen Jiang, Richard Norman, Rosalie Viney, Deborah Street, Nancy Devlin and Mulhern Brendan in Medical Decision Making

sj-docx-5-mdm-10.1177_0272989X251407950 – Supplemental material for Valuation of the EQ-5D-Y-5L Using DCE Methods That Account for Nonlinear Time Preferences Supplemental material, sj-docx-5-mdm-10.1177_0272989X251407950 for Valuation of the EQ-5D-Y-5L Using DCE Methods That Account for Nonlinear Time Preferences by Alice Yu, Bram Roudijk, Peiwen Jiang, Richard Norman, Rosalie Viney, Deborah Street, Nancy Devlin and Mulhern Brendan in Medical Decision Making

sj-docx-6-mdm-10.1177_0272989X251407950 – Supplemental material for Valuation of the EQ-5D-Y-5L Using DCE Methods That Account for Nonlinear Time Preferences Supplemental material, sj-docx-6-mdm-10.1177_0272989X251407950 for Valuation of the EQ-5D-Y-5L Using DCE Methods That Account for Nonlinear Time Preferences by Alice Yu, Bram Roudijk, Peiwen Jiang, Richard Norman, Rosalie Viney, Deborah Street, Nancy Devlin and Mulhern Brendan in Medical Decision Making

sj-docx-7-mdm-10.1177_0272989X251407950 – Supplemental material for Valuation of the EQ-5D-Y-5L Using DCE Methods That Account for Nonlinear Time Preferences Supplemental material, sj-docx-7-mdm-10.1177_0272989X251407950 for Valuation of the EQ-5D-Y-5L Using DCE Methods That Account for Nonlinear Time Preferences by Alice Yu, Bram Roudijk, Peiwen Jiang, Richard Norman, Rosalie Viney, Deborah Street, Nancy Devlin and Mulhern Brendan in Medical Decision Making

sj-docx-8-mdm-10.1177_0272989X251407950 – Supplemental material for Valuation of the EQ-5D-Y-5L Using DCE Methods That Account for Nonlinear Time Preferences Supplemental material, sj-docx-8-mdm-10.1177_0272989X251407950 for Valuation of the EQ-5D-Y-5L Using DCE Methods That Account for Nonlinear Time Preferences by Alice Yu, Bram Roudijk, Peiwen Jiang, Richard Norman, Rosalie Viney, Deborah Street, Nancy Devlin and Mulhern Brendan in Medical Decision Making

sj-docx-9-mdm-10.1177_0272989X251407950 – Supplemental material for Valuation of the EQ-5D-Y-5L Using DCE Methods That Account for Nonlinear Time Preferences Supplemental material, sj-docx-9-mdm-10.1177_0272989X251407950 for Valuation of the EQ-5D-Y-5L Using DCE Methods That Account for Nonlinear Time Preferences by Alice Yu, Bram Roudijk, Peiwen Jiang, Richard Norman, Rosalie Viney, Deborah Street, Nancy Devlin and Mulhern Brendan in Medical Decision Making

sj-docx-10-mdm-10.1177_0272989X251407950 – Supplemental material for Valuation of the EQ-5D-Y-5L Using DCE Methods That Account for Nonlinear Time Preferences Supplemental material, sj-docx-10-mdm-10.1177_0272989X251407950 for Valuation of the EQ-5D-Y-5L Using DCE Methods That Account for Nonlinear Time Preferences by Alice Yu, Bram Roudijk, Peiwen Jiang, Richard Norman, Rosalie Viney, Deborah Street, Nancy Devlin and Mulhern Brendan in Medical Decision Making

sj-docx-11-mdm-10.1177_0272989X251407950 – Supplemental material for Valuation of the EQ-5D-Y-5L Using DCE Methods That Account for Nonlinear Time Preferences Supplemental material, sj-docx-11-mdm-10.1177_0272989X251407950 for Valuation of the EQ-5D-Y-5L Using DCE Methods That Account for Nonlinear Time Preferences by Alice Yu, Bram Roudijk, Peiwen Jiang, Richard Norman, Rosalie Viney, Deborah Street, Nancy Devlin and Mulhern Brendan in Medical Decision Making

## Notes

i. https://developers.google.com/recaptcha/docs/v3.
