# Supplementary material for: Valuation of the EQ-5D-Y-5L Using DCE Methods That Account for Nonlinear Time Preferences
Source: Med Decis Making. 2026 Jan 13;46(3):343–54. doi: 10.1177/0272989X251407950 (PMC12976102; doi:10.1177/0272989X251407950)
Supplement: sj-docx-1-mdm-10.1177_0272989X251407950 – Supplemental material for Valuation of the EQ-5D-Y-5L Using DCE Methods That Account for Nonlinear Time Preferences [file sj-docx-1-mdm-10.1177_0272989X251407950.docx]

**Appendix A Survey overview**

When respondents entered the survey, they were provided with information about the study and asked to consent to participate. Those who consented were then asked about their age, gender and postcode to determine their eligibility for the study as quotas were in place to ensure a representative Australian sample. Respondents were also asked about whether they have ever had children although this was not used for the quotas. This was followed by self-reporting their health on the EQ-5D-Y-5L questionnaire prior to a short tutorial on the DCE choice tasks. Respondents were then shown a short tutorial on the DCE choice tasks, before being asked to complete DCE choice tasks. The perspective respondents saw in the tutorial and DCE choice tasks depended on whether they were part of Arm 1 or Arm 2. After completion of the DCE choice tasks, respondents were asked some further follow up questions about the DCE choice tasks, and additional demographic and health questions. They were also asked to complete the EQ-5D-5L questionnaire. Respondents were given space to provide free text comments before the conclusion of the survey.
