# Supplementary figures and images for: Valuation of the EQ-5D-Y-5L Using DCE Methods That Account for Nonlinear Time Preferences
Source: Med Decis Making. 2026 Jan 13;46(3):343–54. doi: 10.1177/0272989X251407950 (PMC12976102; doi:10.1177/0272989X251407950)

**Appendix B Tutorial pages in survey: 'self' perspective**


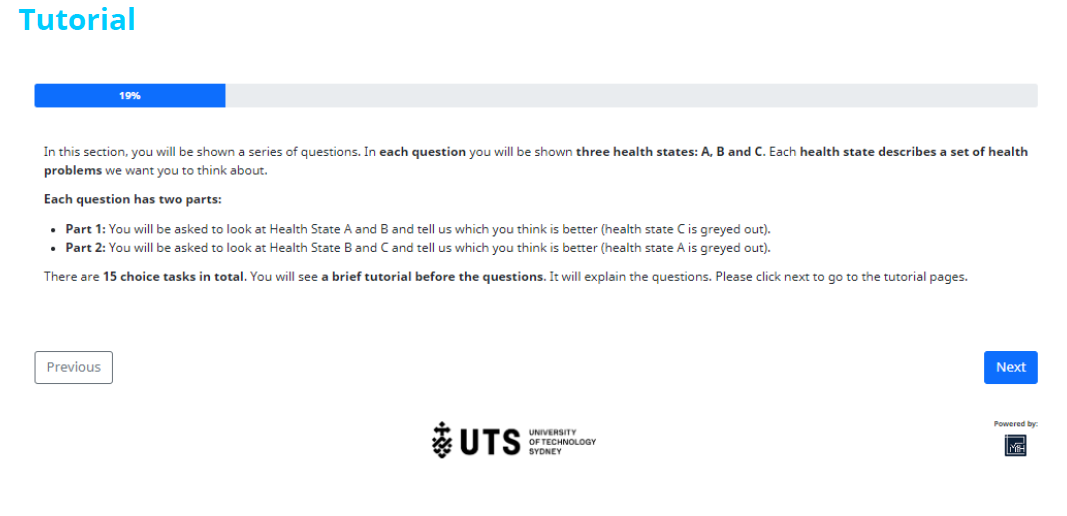


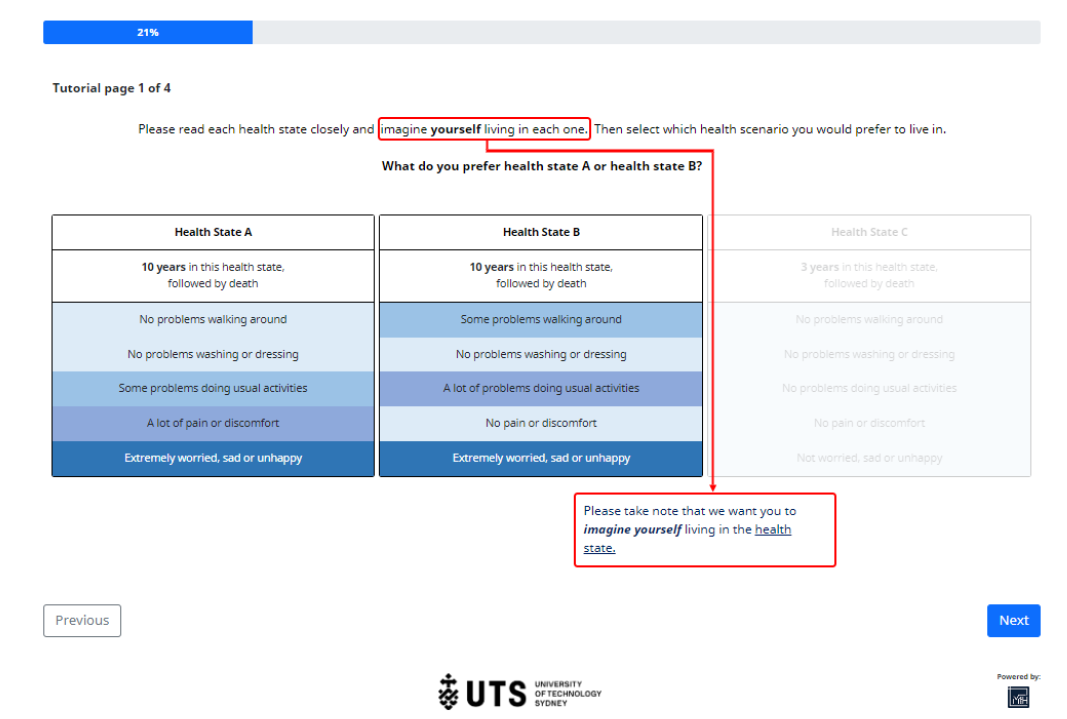


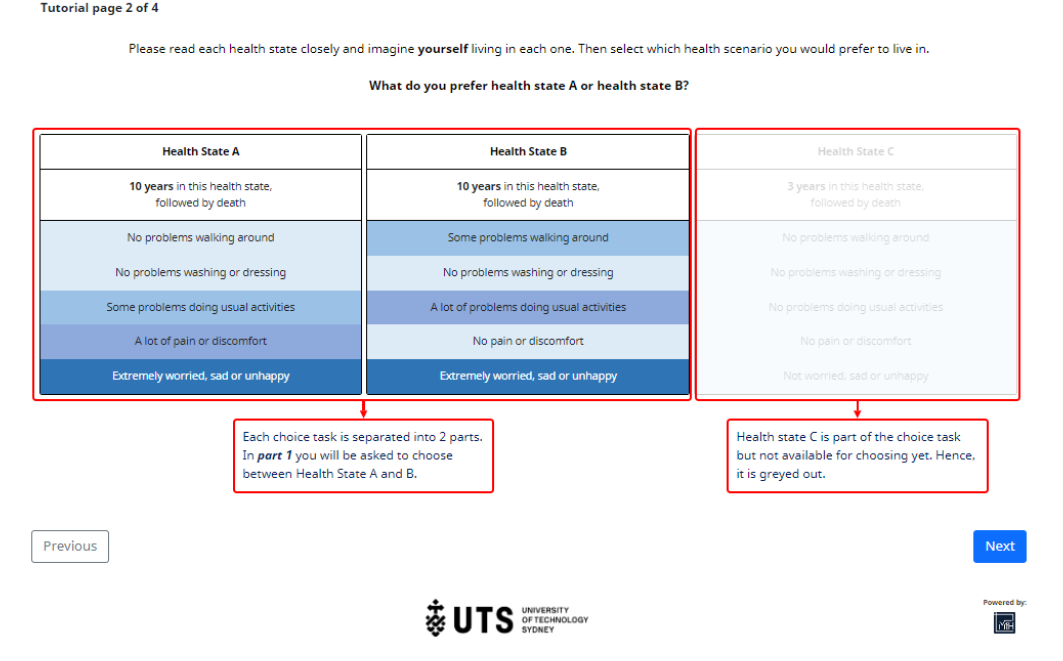


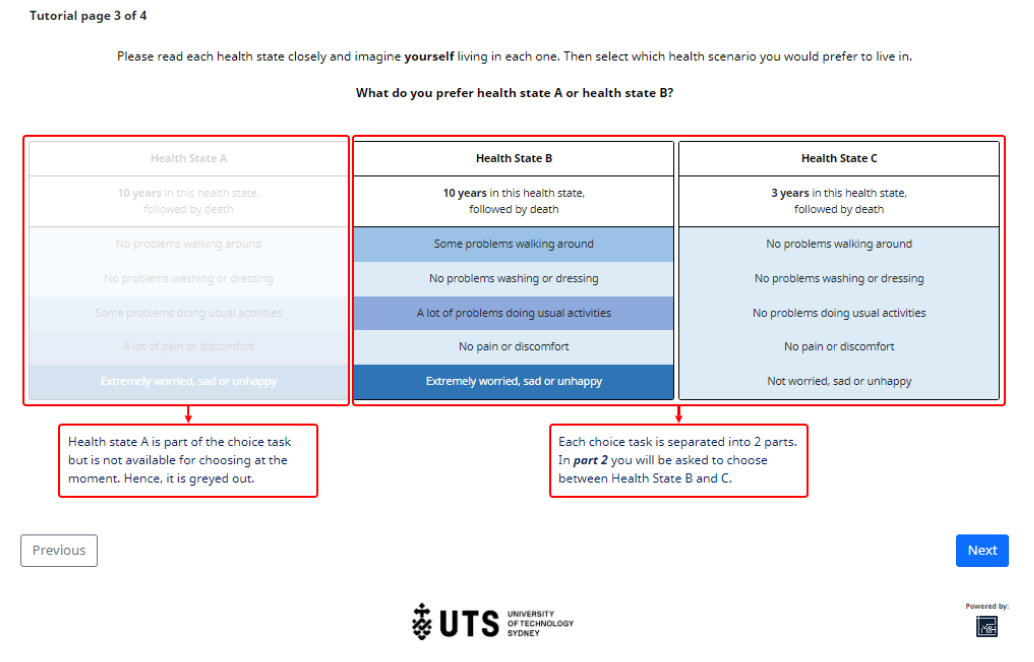


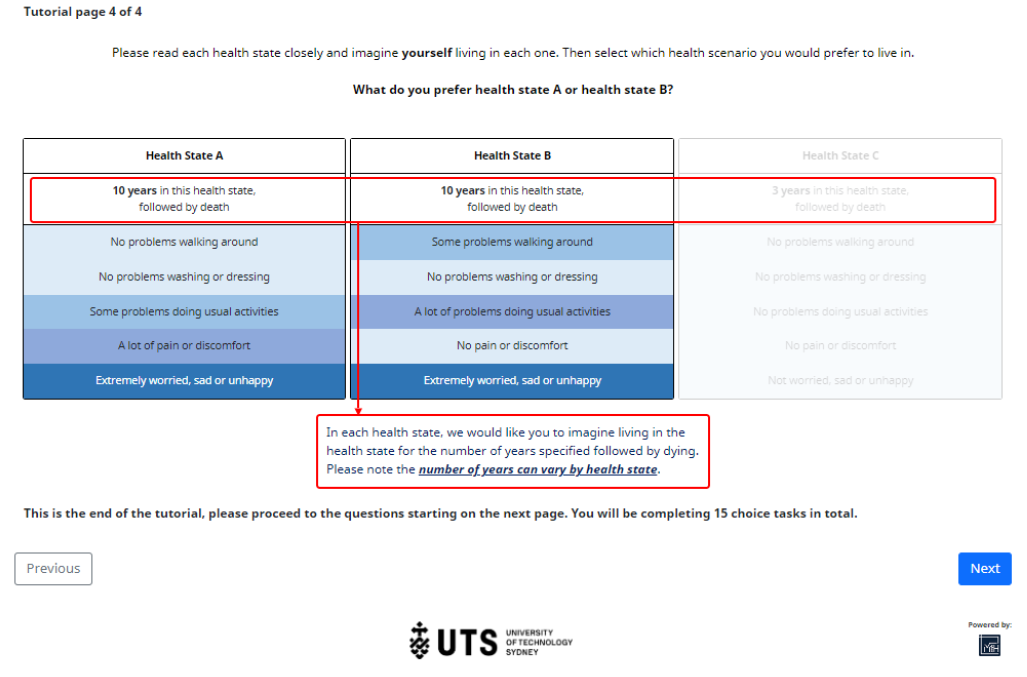

Supplement: sj-docx-2-mdm-10.1177_0272989X251407950 – Supplemental material for Valuation of the EQ-5D-Y-5L Using DCE Methods That Account for Nonlinear Time Preferences [file sj-docx-2-mdm-10.1177_0272989X251407950.docx]
