# Supplementary material for: Valuation of the EQ-5D-Y-5L Using DCE Methods That Account for Nonlinear Time Preferences
Source: Med Decis Making. 2026 Jan 13;46(3):343–54. doi: 10.1177/0272989X251407950 (PMC12976102; doi:10.1177/0272989X251407950)
Supplement: sj-docx-3-mdm-10.1177_0272989X251407950 – Supplemental material for Valuation of the EQ-5D-Y-5L Using DCE Methods That Account for Nonlinear Time Preferences [file sj-docx-3-mdm-10.1177_0272989X251407950.docx]

**Appendix C Bayesian design optimisation process**

The Bayesian efficient design was optimised for a discount rate using an exponential function i.e. nonlinear preferences for time were explicitly considered in the design of the DCE choice tasks. Each Bayesian efficient design included 10 sub-designs i.e. 10 versions each of 15 DCE choice tasks, with respondents randomly assigned to one sub-design. The use of sub-designs increases the robustness and efficiency of the overall DCE design (17, 24). The selection of DCE choice task health states and durations were based on minimising the weighted average Bayesian D-efficiency of the design, with one quarter of the weight assigned to the combined D-efficiency and three quarters of the weight assigned to the individual D-efficiencies of the sub-designs. More weight was given to the D-efficiencies of the sub-designs to ensure more power at the individual level rather than the aggregate levels. This is relevant for mixed logit (MXL) models where individual parameters are estimated.
