# Supplementary material for: Valuation of the EQ-5D-Y-5L Using DCE Methods That Account for Nonlinear Time Preferences
Source: Med Decis Making. 2026 Jan 13;46(3):343–54. doi: 10.1177/0272989X251407950 (PMC12976102; doi:10.1177/0272989X251407950)
Supplement: sj-docx-4-mdm-10.1177_0272989X251407950 – Supplemental material for Valuation of the EQ-5D-Y-5L Using DCE Methods That Account for Nonlinear Time Preferences [file sj-docx-4-mdm-10.1177_0272989X251407950.docx]

**Appendix D: Panel provider and data quality issues**

Pure Profile was the panel provider that was ultimately used for recruitment. This was after significant quality issues and delays from the use of panel provider, Cint. There were concerns about data quality issues after the analysis of the first 200 respondents. It was found that while the conditional logit model was able to run, extreme scaling was noted when running MXL models e.g. values of around -4 for state 55555. Strange patterns were also noted in the data. A third of respondents always chose health state C when choosing between health state B and C i.e. flatliners. This could be valid preferences by respondents however, it was noted that 80% of flatliners were from computer/tablet device users while 20% were from mobile device users. This is a large proportion considering a third of respondents were mobile device users.

As a result, more stringent data quality checks were put in place (see description of quality checks). Cint also increased their internal quality criteria to the highest level for recruitment. The next 200 respondents were recruited, but this took 3 weeks to collect. Estimation of the MXL model was still problematic based on the new set of data. It was also not considered feasible in terms of timelines for collection of 2000 respondents in total if the collection of 200 respondents were to take 3 weeks.

The lack of confidence in data collected led the team to the decision to terminate recruitment with Cint and move to a new panel provider. Pure Profile was chosen and results from the first 200 respondents proved to be fruitful. The MXL model was showing reasonable estimations. Recruitment time was also much more reasonable for 200 respondents, taking a couple of days to recruit as opposed to weeks.
