# Supplementary material for: Valuation of the EQ-5D-Y-5L Using DCE Methods That Account for Nonlinear Time Preferences
Source: Med Decis Making. 2026 Jan 13;46(3):343–54. doi: 10.1177/0272989X251407950 (PMC12976102; doi:10.1177/0272989X251407950)
Supplement: sj-docx-5-mdm-10.1177_0272989X251407950 – Supplemental material for Valuation of the EQ-5D-Y-5L Using DCE Methods That Account for Nonlinear Time Preferences [file sj-docx-5-mdm-10.1177_0272989X251407950.docx]

**Appendix E: MXL model results: linear time preferences**

| ‘Self' arm (N = 955) | Mean | SD | L 95%CI | U95% CI | QALY scale values |
| --- | --- | --- | --- | --- | --- |
| Full Health | 0.63 | 0.05 | 0.54 | 0.73 |  |
| MO2xFull Health | -0.05 | 0.01 | -0.07 | -0.04 | -0.09 |
| MO3xFull Health | -0.08 | 0.01 | -0.1 | -0.07 | -0.13 |
| MO4xFull Health | -0.18 | 0.01 | -0.21 | -0.17 | -0.29 |
| MO5xFull Health | -0.35 | 0.02 | -0.38 | -0.32 | -0.55 |
| SC2xFull Health | -0.05 | 0.01 | -0.06 | -0.04 | -0.08 |
| SC3xFull Health | -0.07 | 0.01 | -0.08 | -0.05 | -0.11 |
| SC4xFull Health | -0.17 | 0.01 | -0.19 | -0.15 | -0.26 |
| SC5xFull Health | -0.32 | 0.02 | -0.35 | -0.29 | -0.51 |
| UA2xFull Health | -0.04 | 0.01 | -0.05 | -0.02 | -0.06 |
| UA3xFull Health | -0.06 | 0.01 | -0.08 | -0.04 | -0.1 |
| UA4xFull Health | -0.14 | 0.01 | -0.16 | -0.12 | -0.23 |
| UA5xFull Health | -0.24 | 0.01 | -0.27 | -0.21 | -0.38 |
| PD2xFull Health | -0.06 | 0.01 | -0.07 | -0.04 | -0.09 |
| PD3xFull Health | -0.09 | 0.01 | -0.1 | -0.07 | -0.14 |
| PD4xFull Health | -0.23 | 0.01 | -0.26 | -0.21 | -0.37 |
| PD5xFull Health | -0.45 | 0.02 | -0.5 | -0.41 | -0.71 |
| AD2xFull Health | -0.06 | 0.01 | -0.08 | -0.04 | -0.09 |
| AD3xFull Health | -0.14 | 0.01 | -0.16 | -0.12 | -0.23 |
| AD4xFull Health | -0.2 | 0.01 | -0.22 | -0.18 | -0.32 |
| AD5xFull Health | -0.31 | 0.02 | -0.34 | -0.28 | -0.49 |
| Health state 55555 |  |  |  |  | -1.64 |

| ‘10-year-old' arm (N = 947) | Mean | SD | L 95%CI | U 95% CI | QALY scale values |
| --- | --- | --- | --- | --- | --- |
| Full Health | 0.58 | 0.05 | 0.49 | 0.68 |  |
| MO2xFull Health | -0.05 | 0.01 | -0.07 | -0.04 | -0.09 |
| MO3xFull Health | -0.07 | 0.01 | -0.09 | -0.06 | -0.13 |
| MO4xFull Health | -0.17 | 0.01 | -0.19 | -0.15 | -0.29 |
| MO5xFull Health | -0.26 | 0.01 | -0.29 | -0.23 | -0.44 |
| SC2xFull Health | -0.03 | 0.01 | -0.04 | -0.01 | -0.05 |
| SC3xFull Health | -0.06 | 0.01 | -0.07 | -0.04 | -0.1 |
| SC4xFull Health | -0.12 | 0.01 | -0.13 | -0.1 | -0.2 |
| SC5xFull Health | -0.2 | 0.01 | -0.22 | -0.17 | -0.34 |
| UA2xFull Health | -0.04 | 0.01 | -0.06 | -0.03 | -0.07 |
| UA3xFull Health | -0.07 | 0.01 | -0.08 | -0.05 | -0.11 |
| UA4xFull Health | -0.15 | 0.01 | -0.17 | -0.13 | -0.26 |
| UA5xFull Health | -0.23 | 0.01 | -0.26 | -0.21 | -0.4 |
| PD2xFull Health | -0.07 | 0.01 | -0.09 | -0.05 | -0.12 |
| PD3xFull Health | -0.11 | 0.01 | -0.12 | -0.09 | -0.18 |
| PD4xFull Health | -0.31 | 0.02 | -0.35 | -0.28 | -0.54 |
| PD5xFull Health | -0.62 | 0.03 | -0.68 | -0.56 | -1.07 |
| AD2xFull Health | -0.08 | 0.01 | -0.1 | -0.07 | -0.15 |
| AD3xFull Health | -0.19 | 0.01 | -0.22 | -0.17 | -0.33 |
| AD4xFull Health | -0.28 | 0.02 | -0.31 | -0.25 | -0.48 |
| AD5xFull Health | -0.42 | 0.02 | -0.46 | -0.37 | -0.72 |
| Health state 55555 |  |  |  |  | -1.96 |
