# Supplementary material for: Valuation of the EQ-5D-Y-5L Using DCE Methods That Account for Nonlinear Time Preferences
Source: Med Decis Making. 2026 Jan 13;46(3):343–54. doi: 10.1177/0272989X251407950 (PMC12976102; doi:10.1177/0272989X251407950)
Supplement: sj-docx-6-mdm-10.1177_0272989X251407950 – Supplemental material for Valuation of the EQ-5D-Y-5L Using DCE Methods That Account for Nonlinear Time Preferences [file sj-docx-6-mdm-10.1177_0272989X251407950.docx]

**Appendix F Completed responses by data collection round**

| Data collection | | No. | No. failed traffic light test |
| --- | --- | --- | --- |
| ‘Self' arm (n = 1004) | Round 1 | 211 | 46 |
|  | Round 2 | 322 | 73 |
|  | Round 3 | 205 | 52 |
|  | Round 4 | 266 | 72 |
| ‘10 year old' arm (n = 1011) | Round 1 | 218 | N/A |
|  | Round 2 | 202 | N/A |
|  | Round 3 | 195 | N/A |
|  | Round 4 | 396 | N/A |
