# Supplementary material for: Valuation of the EQ-5D-Y-5L Using DCE Methods That Account for Nonlinear Time Preferences
Source: Med Decis Making. 2026 Jan 13;46(3):343–54. doi: 10.1177/0272989X251407950 (PMC12976102; doi:10.1177/0272989X251407950)
Supplement: sj-docx-7-mdm-10.1177_0272989X251407950 – Supplemental material for Valuation of the EQ-5D-Y-5L Using DCE Methods That Account for Nonlinear Time Preferences [file sj-docx-7-mdm-10.1177_0272989X251407950.docx]

# Appendix G: Correlated MXL Model Raw Parameter Estimates

| Raw Parameter Estimates | ‘Self' arm (N = 955) | | | | ‘10-year-old' arm (N = 947) | | | |
| --- | --- | --- | --- | --- | --- | --- | --- | --- |
|  | Mean | SD | L95%CI | U95%CI | Mean | SD | L95%CI | U95%CI |
| Full Health | 1.95 | 0.1 | 1.76 | 2.14 | 1.56 | 0.08 | 1.39 | 1.72 |
| MO2xFull Health | -0.11 | 0.01 | -0.14 | -0.08 | -0.1 | 0.01 | -0.12 | -0.07 |
| MO3xFull Health | -0.17 | 0.02 | -0.2 | -0.14 | -0.14 | 0.01 | -0.17 | -0.11 |
| MO4xFull Health | -0.42 | 0.02 | -0.47 | -0.38 | -0.35 | 0.02 | -0.39 | -0.31 |
| MO5xFull Health | -0.84 | 0.03 | -0.91 | -0.77 | -0.55 | 0.02 | -0.6 | -0.5 |
| SC2xFull Health | -0.1 | 0.01 | -0.12 | -0.07 | -0.03 | 0.01 | -0.06 | -0.01 |
| SC3xFull Health | -0.13 | 0.01 | -0.16 | -0.1 | -0.09 | 0.01 | -0.12 | -0.07 |
| SC4xFull Health | -0.39 | 0.02 | -0.43 | -0.35 | -0.23 | 0.02 | -0.26 | -0.2 |
| SC5xFull Health | -0.79 | 0.03 | -0.85 | -0.73 | -0.41 | 0.02 | -0.45 | -0.37 |
| UA2xFull Health | -0.05 | 0.01 | -0.08 | -0.03 | -0.05 | 0.01 | -0.08 | -0.03 |
| UA3xFull Health | -0.11 | 0.01 | -0.14 | -0.08 | -0.11 | 0.01 | -0.14 | -0.08 |
| UA4xFull Health | -0.32 | 0.02 | -0.36 | -0.28 | -0.29 | 0.02 | -0.33 | -0.26 |
| UA5xFull Health | -0.58 | 0.03 | -0.63 | -0.53 | -0.49 | 0.02 | -0.53 | -0.45 |
| PD2xFull Health | -0.1 | 0.01 | -0.13 | -0.07 | -0.14 | 0.01 | -0.16 | -0.11 |
| PD3xFull Health | -0.18 | 0.02 | -0.21 | -0.15 | -0.21 | 0.02 | -0.24 | -0.18 |
| PD4xFull Health | -0.56 | 0.03 | -0.61 | -0.51 | -0.69 | 0.03 | -0.75 | -0.63 |
| PD5xFull Health | -1.13 | 0.04 | -1.22 | -1.04 | -1.38 | 0.06 | -1.49 | -1.28 |
| AD2xFull Health | -0.12 | 0.01 | -0.15 | -0.09 | -0.17 | 0.01 | -0.2 | -0.14 |
| AD3xFull Health | -0.32 | 0.02 | -0.36 | -0.29 | -0.41 | 0.02 | -0.45 | -0.37 |
| AD4xFull Health | -0.46 | 0.02 | -0.51 | -0.42 | -0.6 | 0.03 | -0.65 | -0.55 |
| AD5xFull Health | -0.76 | 0.03 | -0.83 | -0.69 | -0.93 | 0.04 | -1.01 | -0.85 |
| Discount rate | 0.17 | 0.01 | 0.15 | 0.18 | 0.15 | 0.01 | 0.13 | 0.16 |
| Log likelihood | -9982 | 109.3 | -10200 | -9768 | -9813 | 119.4 | -10040 | -9576 |
