# Supplementary material for: Valuation of the EQ-5D-Y-5L Using DCE Methods That Account for Nonlinear Time Preferences
Source: Med Decis Making. 2026 Jan 13;46(3):343–54. doi: 10.1177/0272989X251407950 (PMC12976102; doi:10.1177/0272989X251407950)
Supplement: sj-docx-8-mdm-10.1177_0272989X251407950 – Supplemental material for Valuation of the EQ-5D-Y-5L Using DCE Methods That Account for Nonlinear Time Preferences [file sj-docx-8-mdm-10.1177_0272989X251407950.docx]

**Appendix H Summary of respondent demographics**

| Basic demographics | ‘Self' arm No. (%) | ‘10-year-old' arm No. (%) | Population % |
| --- | --- | --- | --- |
| Gender |  |  |  |
| Male | 462 (49%) | 431 (46%) | 49% |
| Female | 485 (50%) | 515 (54%) | 51% |
| Non-binary | 4 (0%) | 1 (0%) |  |
| Prefer not to say | 4 (0%) | N/A |  |
| Age Group |  |  |  |
| 18-24 | 70 (7%) | 82 (9%) | 12% |
| 25-39 | 244 (26%) | 262 (28%) | 28% |
| 40-59 | 334 (35%) | 321 (33%) | 32% |
| 60+ | 307 (32%) | 282 (30%) | 27% |
| State |  |  |  |
| ACT | 18 (2%) | 20 (2%) | 2% |
| NSW | 272 (28%) | 280 (30%) | 31% |
| NT | 3 (0%) | 6 (1%) | 1% |
| QLD | 198 (21%) | 195 (20%) | 20% |
| SA | 75 (8%) | 67 (7%) | 7% |
| TAS | 22 (2%) | 21 (2%) | 2% |
| VIC | 258 (27%) | 247 (26%) | 26% |
| WA | 109 (11%) | 111 (11%) | 11% |
| Parental/caregiver status |  |  |  |
| Yes | 611 (63%) | 592 (64%) | 44% |
| No | 344 (37%) | 354 (36%) | 56% |
| N/A |  | 1 (0%) |  |
