# Supplementary material for: Valuation of the EQ-5D-Y-5L Using DCE Methods That Account for Nonlinear Time Preferences
Source: Med Decis Making. 2026 Jan 13;46(3):343–54. doi: 10.1177/0272989X251407950 (PMC12976102; doi:10.1177/0272989X251407950)
Supplement: sj-docx-9-mdm-10.1177_0272989X251407950 – Supplemental material for Valuation of the EQ-5D-Y-5L Using DCE Methods That Account for Nonlinear Time Preferences [file sj-docx-9-mdm-10.1177_0272989X251407950.docx]

**Appendix I Summary of DCE choice task ratings**

For the ‘self’ arm, some respondents found the DCE choice task difficult with 44% agreeing or strongly agreeing with the statement ‘I found the choice task difficult’. However, 68% of respondents disagreed or strongly disagreed with the statement that they found it difficult to tell the difference between health states i.e. they did not find it difficult although some respondents (46%) agreed or strongly agreed that it was difficult to choose between health states.

In contrast to the ‘self’ arm, many more respondents in the ’10-year-old’ arm, found the DCE choice task difficult with 66% agreeing or strongly agreeing that ‘I found the choice task difficult’. Similar to the ‘self’ arm, while most respondents did not find it difficult to tell the difference between health states (61%), it was difficult to choose between them (67%).

| Rating (%) | Choice task difficult | | Difficult to tell difference between health states | | Difficult to choose between health states | |
| --- | --- | --- | --- | --- | --- | --- |
|  | ‘Self' Arm | ‘10-year-old' arm | ‘Self' Arm | ‘10-year-old' arm | ‘Self' Arm | ‘10-year-old' arm |
| Strongly agree | 10% | 25% | 4% | 4% | 9% | 21% |
| Agree | 34% | 42% | 10% | 16% | 37% | 46% |
| Neutral | 19% | 14% | 18% | 20% | 20% | 14% |
| Disagree | 30% | 16% | 51% | 48% | 26% | 16% |
| Strongly Disagree | 8% | 4% | 18% | 13% | 8% | 3% |
