# Supplementary material for: Valuation of the EQ-5D-Y-5L Using DCE Methods That Account for Nonlinear Time Preferences
Source: Med Decis Making. 2026 Jan 13;46(3):343–54. doi: 10.1177/0272989X251407950 (PMC12976102; doi:10.1177/0272989X251407950)
Supplement: Supplementary file 11 [file sj-docx-11-mdm-10.1177_0272989X251407950.docx]

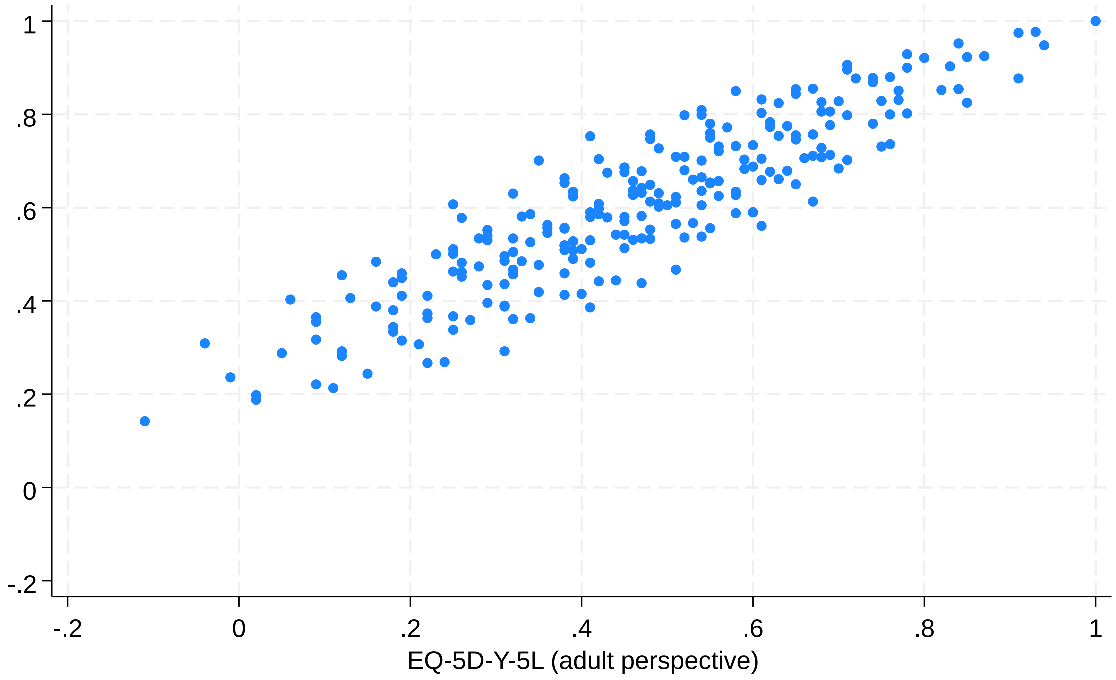
1
.8
.6
.4
.2
0
-.2
-.2
0
.2
.4
.6
.8
1
EQ-5D-Y-5L (adult perspective)

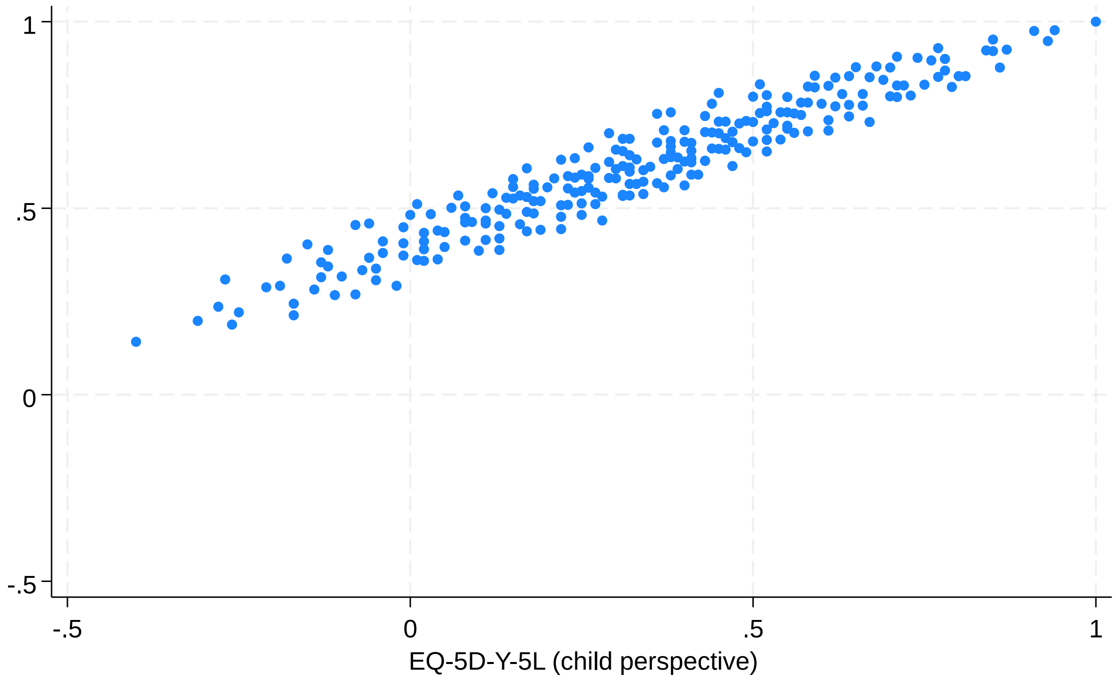
1
.5
0
-.5
-.5
0
.5
1
EQ-5D-Y-5L (child perspective)
